# Supplementary material for: Mixotrophic cultivation of Spirulina platensis in dairy wastewater: Effects on the production of biomass, biochemical composition and antioxidant capacity
Source: PLoS One. 2019 Oct 24;14(10):e0224294. doi: 10.1371/journal.pone.0224294 (PMC6812818; doi:10.1371/journal.pone.0224294)
Supplement: S1 File — (DOCX) [file pone.0224294.s001.docx]

**Supporting Information**

**Figure A**. Curve Standard of dry weight biomass referring to the autotrophic culture (Zarrouk medium).

**Figure B**. Curve Standard of dry weight biomass referring to the mixotrophic culture with 2.5% of cheese whey.

**Figure C.** Curve Standard of dry weight biomass referring to the mixotrophic culture with 5.0% of cheese whey.

**Figure D**. Standard Curve of dry weight biomass referring to the mixotrophic culture with 10.0% of cheese whey.

**Figure E**. Standard curve of Gallic acid used for determination of total phenolics.

**Figure F**. Standard curve of Trolox used for determination of Antioxidant activity of *Spirulina platensis* by the Free Radical Capture method - ABST^•+^

**Figure G**. Standard curve of Trolox used for determination of Antioxidant activity of *Spirulina platensis* by the FRAP.

**Figure H**. Standard curve of Ferrous sulphate used for determination of Antioxidant activity of *Spirulina platensis* by the FRAP.

**Figure I.** Standard curve of glucose used for determination of carbohydrates of *Spirulina platensis.*

**Table A.** Biochemical composition of *S. platensis* biomass grown on autotrophic Zarrouk medium and mixotrophic medium supplemented with buffalo cheese whey at concentrations of 2.5%, 5.0% and 10.0%.

| **% Whey** | **Replication** | **% Dry matter** | **% Total protein** | **% Fat** | **% Carbohydrates** | **% Ash** |
| --- | --- | --- | --- | --- | --- | --- |
| **0** | 1 | 95.47 | 64.38 | 5.06 | 27.94 | 5.89 |
| **0** | 2 | 92.48 | 67.71 | 5.64 | 25.36 | 5.75 |
| **0** | 3 | 93 | 64.55 | 4.84 | 28.21 | 6.47 |
| **Average** |  | **93.65** | **65.55** | 5.18 | **27.17** | **6.04** |
| **Standard deviation** |  | 1.6 | 1.88 | 0.31 | 1.57 | 0.38 |
| **2.5** | 1 | 91.17 | 60.94 | 2.06 | 22.81 | 14.24 |
| **2.5** | 2 | 91.81 | 60.58 | 2.07 | 25.01 | 14.18 |
| **2.5** | 3 | 92.32 | 60.34 | 2.00 | 22.04 | 14.21 |
| **Average** |  | **91.77** | **60.62** | **2.04** | **23.29** | **14.21** |
| **Standard deviation** |  | 0.58 | 0.3 | 0.03 | 1.54 | 0.03 |
| **5** | 1 | 90.21 | 44.73 | 2.71 | 48.7 | 13.96 |
| **5** | 2 | 90.34 | 43.75 | 2.65 | 47.18 | 13.94 |
| **5** | 3 | 91.16 | 45.21 | 2.32 | 47.6 | 14.2 |
| **Average** |  | **90.57** | **44.56** | **2.56** | **47.83** | **14.03** |
| **Standard deviation** |  | 0.52 | 0.74 | 0.16 | 0.78 | 0.14 |
| **10** | 1 | 96.01 | 39.41 | 3.68 | 44.17 | 6.4 |
| **10** | 2 | 94.65 | 39.41 | 3.25 | 36.87 | 6.23 |
| **10** | 3 | 94.09 | 40.04 | 3.28 | 40.9 | 6.45 |
| **Average** |  | **94.92** | **39.62** | **3.40** | **40.65** | **6.36** |
| **Standard deviation** |  | 0.99 | 0.36 | 0.18 | 3.66 | 0.12 |

**Table B.** Phenolic compounds content of *S. platensis* biomass grown on autotrophic Zarrouk medium and mixotrophic medium supplemented with buffalo cheese whey at concentrations of 2.5%, 5.0% and 10.0%.

|  | **% Whey** | | | |
| --- | --- | --- | --- | --- |
|  | **0** | **2.5** | **5** | **10** |
| **Phenolic (mg Galic acid/g)** | 32.16 | 30.85 | 32.87 | 22.51 |
|  | 33.53 | 30.85 | 33.58 | 22.39 |
|  | 33.9 | 30.1 | 33.24 | 22.22 |
| **Average** | **33.2** | **30.6** | **33.23** | **22.37** |
| **Standard deviation** | 0.92 | 0.43 | 0.36 | 0.14 |

**Table C.** Antioxidant capacity compounds content of *S. platensis* biomass grown on autotrophic Zarrouk medium and mixotrophic medium supplemented with buffalo cheese whey at concentrations of 2.5%, 5.0% and 10.0%.

|  |  | **Antioxidant capacity** | |  |  |
| --- | --- | --- | --- | --- | --- |
| **% Whey** | **Replication** | **FRAP (µmol SF/g)** | **FRAP (µmol Tx/g)** | **ABTS (µmol Tx/g)** | |
| 0 | 1 | 35.35 | 20.68 | 18.1 | |
| 0 | 2 | 34.48 | 20.17 | 17.09 | |
| 0 | 3 | 36.23 | 21.19 | 17.83 | |
| **Average** |  | **35.35** | **20.68** | **17.67** | |
| 2.5 | 1 | 15.25 | 8.95 | 14.73 | |
| 2.5 | 2 | 15.32 | 8.99 | 14.8 | |
| 2.5 | 3 | 15.17 | 8.9 | 15 | |
| **Average** |  | **15.25** | **8.95** | **14.84** | |
| 5 | 1 | 15.39 | 9.03 | 14.73 | |
| 5 | 2 | 15.49 | 9.09 | 14.13 | |
| 5 | 3 | 15.28 | 8.97 | 15 | |
| **Average** |  | **15.39** | **9.03** | **14.62** | |
| 10 | 1 | 14.54 | 8.54 | 11.64 | |
| 10 | 2 | 14.68 | 8.62 | 11.57 | |
| 10 | 3 | 14.4 | 8.45 | 11.84 | |
| **Average** |  | **14.54** | **8.54** | **11.68** | |

**Table D.** Profile of the fatty acid methyl esters of *S. platensis* biomass grown on autotrophic Zarrouk medium and mixotrophic medium supplemented with buffalo cheese whey at concentrations of 2.5%, 5.0% and 10.0%.

| **Profile of the fatty acid methyl esters (% area)** | | | | | | | | |
| --- | --- | --- | --- | --- | --- | --- | --- | --- |
| **% Whey** | **0** | **0** | **2.5** | **2.5** | **5.0** | **5.0** | **10.0** | **10.0** |
| *C10:0* |  |  |  |  | 0.722 | 0.699 | 0.694 | 0.625 |
| *C12:0* | 1.11 | 0.975 | 0.753 | 0.728 | 0.641 | 0.604 | 0.65 | 0.625 |
| *C13:0* | 1.513 | 1.431 | 1.03 | 1.017 | 0.925 | 0.744 | 0.938 | 0.921 |
| *C14:0* | 0.621 | 1.395 | 0.707 | 0.724 | 0.59 | 0.593 | 0.242 | 0.23 |
| *C14:1* | 0.897 | 0.965 | 0.692 | 0.693 | 0.484 | 0.483 | 0.512 | 0.504 |
| *C15:0* | 0.556 | 0.298 | 0.321 | 0.317 | 0.3 | 0.287 | 0.306 | 0.301 |
| *C15:1* | 0.678 | 0.663 | 0.617 | 0.622 | 0.599 | 0.582 | 0.67 | 0.506 |
| *C16:0* | 40.599 | 42.153 | 45.752 | 45.72 | 44.91 | 44.871 | 44.29 | 43.681 |
| *C16:1* | 1.715 | 1.668 | 2.235 | 2.204 | 2.311 | 2.294 | 2.879 | 2.825 |
| *C17:0* | 0.277 | 0.16 | 0.491 | 0.499 | 0.351 | 0.187 | 0.355 | 0.165 |
| *C17:1* | 0.284 | 0.253 | 0.223 | 0.231 |  | 0.375 | 2.418 | 2.519 |
| *C18:0* | 4.422 | 2.307 | 2.217 | 2.396 | 2.455 | 2.555 | 0.242 | 0.245 |
| *C18:1* | nd | nd | 0.633 | 0.674 | 0.69 | 0.741 | 5.232 | 0.679 |
| *C18:1n9c* | 9.783 | 11.382 | 10.913 | 10.921 | 12.009 | 12.061 | 3.254 | 3.259 |
| *C18:1n9t* | nd | nd | 0.346 | 0.366 | nd | nd | nd | nd |
| *C18:2n6c* | 11.589 | 10.991 | 10.462 | 10.449 | 8.871 | 8.846 | 10.68 | 11.237 |
| *C18:2n6t* | nd | nd | nd | nd | nd | nd | nd | nd |
| *C20:0* | 15.148 | 14.539 | 16.098 | 16.129 | 17.25 | 17.298 | 19.881 | 19.892 |
| *C20:2* | 0.19 | 0.159 | nd | nd | nd | nd | nd | nd |
| *C20:3n6* | 0.594 | 0.569 | 0.205 | 0.21 | 0.435 | 0.443 | nd | nd |
|  |  |  |  |  |  |  |  |  |

**Table E.** pH of *S. platensis* grown on autotrophic Zarrouk.

| **% 0.0** | | | | | |
| --- | --- | --- | --- | --- | --- |
| **Time (day)** | **pH** | | | **Average** | **St. deviation** |
| 0 | 9.2 | 9.3 | 9.2 | 9.23 | 0.05 |
| 1 | 9.7 | 9.8 | 9.7 | 9.73 | 0.05 |
| 2 | 9.9 | 9.9 | 9.9 | 9.90 | 0.00 |
| 3 | 9.6 | 9.7 | 9.7 | 9.67 | 0.05 |
| 4 | 9.8 | 9.8 | 9.9 | 9.83 | 0.05 |
| 5 | 9.9 | 10 | 10.1 | 10.00 | 0.08 |
| 7 | 10 | 10 | 10 | 10.00 | 0.00 |
| 8 | 10.1 | 10.1 | 10.2 | 10.13 | 0.05 |
| 9 | 10.2 | 10.2 | 10.3 | 10.23 | 0.05 |
| 10 | 10.3 | 10.2 | 10.4 | 10.30 | 0.08 |
| 11 | 10.4 | 10.2 | 10.4 | 10.33 | 0.09 |
| 12 | 10.4 | 10.4 | 10.5 | 10.43 | 0.05 |
| 14 | 10.8 | 10.8 | 11 | 10.87 | 0.09 |
| 15 | 11 | 11 | 11.3 | 11.10 | 0.14 |
| 16 | 11.3 | 11.2 | 11.6 | 11.37 | 0.17 |
| 17 | 11.8 | 11.6 | 12 | 11.80 | 0.16 |

**Table F.** pH of *S. platensis* grown on mixotrophic medium supplemented with buffalo cheese whey at concentrations of 2.5%.

| **% 2.5** | | | | | |
| --- | --- | --- | --- | --- | --- |
| **Time (day)** | **pH** | | | **Average** | **St. deviation** |
| 0 | 9.3 | 9.4 | 9.3 | 9.33 | 0.05 |
| 1 | 9.6 | 9.6 | 9.5 | 9.57 | 0.05 |
| 2 | 9.4 | 9.1 | 9.2 | 9.23 | 0.12 |
| 3 | 9.4 | 9.3 | 9.4 | 9.37 | 0.05 |
| 4 | 9.5 | 9.6 | 9.5 | 9.53 | 0.05 |
| 6 | 9.5 | 9.7 | 9.7 | 9.63 | 0.09 |
| 7 | 9.8 | 9.9 | 9.9 | 9.87 | 0.05 |
| 8 | 10.2 | 10.1 | 10 | 10.10 | 0.08 |
| 9 | 9.9 | 10 | 10 | 9.97 | 0.05 |
| 10 | 10 | 10.1 | 10.1 | 10.07 | 0.05 |
| 11 | 10 | 10.1 | 10.1 | 10.07 | 0.05 |
| 13 | 10.2 | 10.4 | 10.4 | 10.33 | 0.09 |
| 14 | 10.4 | 10.6 | 10.5 | 10.50 | 0.08 |
| 15 | 10.4 | 10.6 | 10.5 | 10.50 | 0.08 |
| 16 | 10.2 | 10.6 | 10.6 | 10.47 | 0.19 |
| 17 | 10.3 | 10.6 | 10.6 | 10.50 | 0.14 |

**Table G.** pH of *S. platensis* biomass grown on mixotrophic medium supplemented with buffalo cheese whey at concentrations of 5.0%.

| **% 5.0** | | | | | |
| --- | --- | --- | --- | --- | --- |
| **Time (day)** | **pH** | | | **Average** | **St. deviation** |
| 0 | 9.3 | 9.4 | 9.4 | 9.37 | 0.05 |
| 1 | 9.6 | 9.7 | 9.6 | 9.63 | 0.05 |
| 2 | 8.8 | 9.2 | 9.3 | 9.10 | 0.22 |
| 3 | 9.4 | 9.4 | 9.5 | 9.43 | 0.05 |
| 5 | 9.3 | 9.6 | 9.8 | 9.57 | 0.21 |
| 6 | 9.6 | 9.8 | 9.9 | 9.77 | 0.12 |
| 7 | 9.9 | 10 | 10 | 9.97 | 0.05 |
| 8 | 9.8 | 10 | 10 | 9.93 | 0.09 |
| 9 | 9.9 | 10 | 10.2 | 10.03 | 0.12 |
| 10 | 10.1 | 10.2 | 10.2 | 10.17 | 0.05 |
| 12 | 10.3 | 10.4 | 10.4 | 10.37 | 0.05 |
| 13 | 10.4 | 10.6 | 10.6 | 10.53 | 0.09 |
| 14 | 10.4 | 10.7 | 10.6 | 10.57 | 0.12 |
| 15 | 10.7 | 10.7 | 10.7 | 10.70 | 0.00 |
| 16 | 10.8 | 10.9 | 10.8 | 10.83 | 0.05 |
| 17 | 11.1 | 11 | 10.9 | 11.00 | 0.08 |

**Table H.** pH of *S. platensis* biomass grown on mixotrophic medium supplemented with buffalo cheese whey at concentrations of 10.0%.

| **%10.0** | | | | | |
| --- | --- | --- | --- | --- | --- |
| **Time (day)** | **pH** | | | **Average** | **St. deviation** |
| 0 | 8.9 | 8.8 | 9.1 | 8.9 | 0.12 |
| 1 | 9 | 9 | 8.9 | 9.0 | 0.05 |
| 2 | 9 | 9.1 | 9.1 | 9.1 | 0.05 |
| 3 | 9.3 | 9.3 | 9.3 | 9.3 | 0.00 |
| 5 | 9.4 | 9.4 | 9.5 | 9.4 | 0.05 |
| 6 | 9.5 | 9.6 | 9.6 | 9.6 | 0.05 |
| 7 | 9.6 | 9.7 | 9.7 | 9.7 | 0.05 |
| 8 | 9.8 | 9.9 | 9.8 | 9.8 | 0.05 |
| 9 | 9.8 | 9.9 | 9.8 | 9.8 | 0.05 |
| 10 | 9.8 | 9.9 | 9.8 | 9.8 | 0.05 |
| 12 | 9.9 | 10 | 10 | 10.0 | 0.05 |
| 13 | 10 | 10.1 | 10 | 10.0 | 0.05 |
| 14 | 10 | 10.1 | 10.1 | 10.1 | 0.05 |
| 15 | 10.2 | 10.2 | 10.2 | 10.2 | 0.00 |
| 16 | 10.2 | 10.2 | 10.2 | 10.2 | 0.00 |
| 17 | 10.2 | 10.3 | 10.3 | 10.3 | 0.05 |

**Table I.** *S. platensis* dry biomass (g/L) grown on autotrophic medium.

| **% 0.0 of Whey** | | | | | |
| --- | --- | --- | --- | --- | --- |
| **Time (day)** | **(mg/ml )*FD** | **(mg/ml )*FD** | **(mg/ml )*FD** | **biomass Average(g/L)** | **SD** |
| 0 | 0,12 | 0,14 | 0,13 | 0,13 | 0,01 |
| 1 | 0,24 | 0,31 | 0,22 | 0,26 | 0,04 |
| 2 | 0,38 | 0,45 | 0,36 | 0,40 | 0,04 |
| 3 | 0,54 | 0,59 | 0,51 | 0,55 | 0,03 |
| 4 | 0,48 | 0,51 | 0,46 | 0,49 | 0,02 |
| 5 | 0,55 | 0,58 | 0,55 | 0,56 | 0,02 |
| 6 | 0,73 | 0,74 | 0,75 | 0,74 | 0,01 |
| 7 | 0,97 | 0,89 | 0,97 | 0,94 | 0,04 |
| 8 | 0,98 | 1,06 | 1,05 | 1,03 | 0,04 |
| 9 | 1,14 | 1,22 | 1,20 | 1,19 | 0,04 |
| 10 | 1,01 | 1,24 | 0,96 | 1,07 | 0,12 |
| 11 | 1,22 | 1,43 | 1,24 | 1,29 | 0,09 |
| 12 | 1,12 | 1,34 | 1,28 | 1,25 | 0,09 |
| 13 | 1,34 | 1,33 | 1,34 | 1,34 | 0,01 |
| 14 | 1,62 | 1,57 | 1,59 | 1,59 | 0,02 |
| 15 | 1,69 | 1,78 | 1,74 | 1,74 | 0,04 |
| 16 | 1,77 | 1,82 | 1,66 | 1,75 | 0,07 |
| 17 | 1,38 | 1,84 | 1,75 | 1,65 | 0,20 |

**Table J.** *S. platensis* dry biomass (g/L) grown on mixotrophic medium supplemented with buffalo cheese whey at concentrations of 2.5%.

| **% 2.5 of Whey** | | | | | |
| --- | --- | --- | --- | --- | --- |
| **Time (day)** | **(mg/ml )*FD** | **(mg/ml )*FD** | **(mg/ml )*FD** | **biomass Average(g/L)** | **Sd** |
| 0 | 0,125 | 0,126 | 0,130 | 0,127 | 0,002 |
| 1 | 0,208 | 0,311 | 0,268 | 0,263 | 0,042 |
| 2 | 0,341 | 0,516 | 0,485 | 0,447 | 0,076 |
| 3 | 0,654 | 0,702 | 0,590 | 0,649 | 0,046 |
| 4 | 0,863 | 0,702 | 0,686 | 0,750 | 0,080 |
| 5 | 0,991 | 1,039 | 0,911 | 0,980 | 0,053 |
| 6 | 1,119 | 0,922 | 1,039 | 1,027 | 0,081 |
| 7 | 0,877 | 1,074 | 1,185 | 1,046 | 0,127 |
| 8 | 1,034 | 1,129 | 1,312 | 1,158 | 0,115 |
| 9 | 1,036 | 1,209 | 1,248 | 1,164 | 0,092 |
| 10 | 1,019 | 1,292 | 1,398 | 1,236 | 0,160 |
| 11 | 1,196 | 1,413 | 0,972 | 1,194 | 0,180 |
| 12 | 1,264 | 1,261 | 1,240 | 1,255 | 0,011 |
| 13 | 1,395 | 1,693 | 1,658 | 1,582 | 0,133 |
| 14 | 1,635 | 1,834 | 2,037 | 1,835 | 0,164 |
| 15 | 1,841 | 1,937 | 2,043 | 1,940 | 0,083 |
| 16 | 2,184 | 2,046 | 2,069 | 2,100 | 0,060 |
| 17 | 2,187 | 2,143 | 2,149 | 2,160 | 0,020 |

**Table K.** *S. platensis* dry biomass (g/L) grown on mixotrophic medium supplemented with buffalo cheese whey at concentrations of 5.0%.

| **% 5.0 of Whey** | | | | | |
| --- | --- | --- | --- | --- | --- |
| **Time (day)** | **(mg/ml )*FD1** | **(mg/ml )*FD2** | **(mg/ml )*FD3** | **biomass Average(g/L)** | **Sd** |
| 0 | 0,13 | 0,11 | 0,12 | 0,12 | 0,01 |
| 1 | 0,47 | 0,50 | 0,39 | 0,46 | 0,05 |
| 2 | 1,07 | 1,42 | 1,32 | 1,27 | 0,15 |
| 3 | 1,34 | 1,41 | 1,31 | 1,35 | 0,04 |
| 4 | 1,43 | 1,45 | 1,40 | 1,43 | 0,02 |
| 5 | 1,59 | 1,67 | 1,51 | 1,59 | 0,06 |
| 6 | 1,61 | 1,69 | 1,46 | 1,59 | 0,10 |
| 7 | 1,74 | 1,58 | 1,75 | 1,69 | 0,08 |
| 8 | 1,87 | 1,89 | 1,79 | 1,85 | 0,04 |
| 9 | 2,10 | 2,01 | 1,94 | 2,02 | 0,07 |
| 10 | 2,20 | 2,09 | 1,90 | 2,06 | 0,12 |
| 11 | 2,15 | 2,17 | 2,13 | 2,15 | 0,02 |
| 12 | 2,40 | 2,30 | 2,25 | 2,32 | 0,06 |
| 13 | 2,58 | 2,78 | 2,66 | 2,67 | 0,08 |
| 14 | 2,87 | 2,91 | 2,65 | 2,81 | 0,12 |
| 15 | 3,02 | 2,90 | 2,75 | 2,89 | 0,11 |
| 16 | 3,13 | 2,97 | 2,87 | 2,99 | 0,11 |
| 17 | 3,06 | 2,95 | 2,86 | 2,96 | 0,08 |

**Table L.** *S. platensis* dry biomass (g/L) grown on mixotrophic medium supplemented with buffalo cheese whey at concentrations of 10.0%.

| **% 10.0 of Whey** | | | | | |
| --- | --- | --- | --- | --- | --- |
| **Time (day)** | **(mg/ml )*FD1** | **(mg/ml )*FD2** | **(mg/ml )*FD3** | **biomass Average(g/L)** | **Sd** |
| 0 | 0,12 | 0,12 | 0,13 | 0,12 | 0,00 |
| 1 | 0,47 | 0,46 | 0,49 | 0,47 | 0,01 |
| 2 | 1,76 | 1,69 | 1,77 | 1,74 | 0,03 |
| 3 | 1,89 | 1,89 | 1,92 | 1,90 | 0,02 |
| 4 | 1,94 | 1,92 | 1,92 | 1,93 | 0,01 |
| 5 | 1,97 | 1,88 | 2,01 | 1,95 | 0,06 |
| 6 | 2,36 | 1,86 | 2,06 | 2,09 | 0,21 |
| 7 | 1,82 | 1,73 | 2,09 | 1,88 | 0,16 |
| 8 | 1,95 | 2,62 | 2,62 | 2,40 | 0,32 |
| 9 | 2,01 | 1,99 | 1,96 | 1,99 | 0,02 |
| 10 | 2,02 | 1,94 | 2,12 | 2,03 | 0,07 |
| 11 | 2,15 | 2,14 | 2,17 | 2,15 | 0,01 |
| 12 | 2,12 | 2,04 | 2,12 | 2,10 | 0,04 |
| 13 | 2,15 | 2,11 | 2,28 | 2,18 | 0,07 |
| 14 | 2,04 | 2,14 | 2,25 | 2,15 | 0,09 |
| 15 | 2,05 | 2,29 | 2,22 | 2,19 | 0,10 |
| 16 | 2,12 | 2,05 | 2,55 | 2,24 | 0,22 |
| 17 | 1,96 | 2,42 | 2,15 | 2,18 | 0,19 |

**Statistical Analyses**

**Xmax**

General Descriptive Statistics

The MEANS Procedure

Coeff of

Variable N Mean Std Dev Variation Minimum Maximum

ƒƒƒƒƒƒƒƒƒƒƒƒƒƒƒƒƒƒƒƒƒƒƒƒƒƒƒƒƒƒƒƒƒƒƒƒƒƒƒƒƒƒƒƒƒƒƒƒƒƒƒƒƒƒƒƒƒƒƒƒƒƒƒƒƒƒƒƒƒƒƒƒƒƒƒƒƒƒƒƒƒƒƒƒƒƒƒƒƒƒƒƒƒƒ

trat 12 10.0000000 9.7700842 97.7008421 0 25.0000000

Xmax 12 2.2374167 0.5100306 22.7955110 1.3790000 3.0640000

ƒƒƒƒƒƒƒƒƒƒƒƒƒƒƒƒƒƒƒƒƒƒƒƒƒƒƒƒƒƒƒƒƒƒƒƒƒƒƒƒƒƒƒƒƒƒƒƒƒƒƒƒƒƒƒƒƒƒƒƒƒƒƒƒƒƒƒƒƒƒƒƒƒƒƒƒƒƒƒƒƒƒƒƒƒƒƒƒƒƒƒƒƒƒ

-------------------------------------------- **trat=0** --------------------------------------------

The MEANS Procedure

Analysis Variable : Xmax

Coeff of

N Mean Std Dev Variation Minimum Maximum

ƒƒƒƒƒƒƒƒƒƒƒƒƒƒƒƒƒƒƒƒƒƒƒƒƒƒƒƒƒƒƒƒƒƒƒƒƒƒƒƒƒƒƒƒƒƒƒƒƒƒƒƒƒƒƒƒƒƒƒƒƒƒƒƒƒƒƒƒƒƒƒƒƒƒƒƒƒƒƒƒƒƒ

3 1.6536667 0.2419036 14.6283146 1.3790000 1.8350000

ƒƒƒƒƒƒƒƒƒƒƒƒƒƒƒƒƒƒƒƒƒƒƒƒƒƒƒƒƒƒƒƒƒƒƒƒƒƒƒƒƒƒƒƒƒƒƒƒƒƒƒƒƒƒƒƒƒƒƒƒƒƒƒƒƒƒƒƒƒƒƒƒƒƒƒƒƒƒƒƒƒƒ

-------------------------------------------- **trat=5** --------------------------------------------

Analysis Variable : Xmax

Coeff of

N Mean Std Dev Variation Minimum Maximum

ƒƒƒƒƒƒƒƒƒƒƒƒƒƒƒƒƒƒƒƒƒƒƒƒƒƒƒƒƒƒƒƒƒƒƒƒƒƒƒƒƒƒƒƒƒƒƒƒƒƒƒƒƒƒƒƒƒƒƒƒƒƒƒƒƒƒƒƒƒƒƒƒƒƒƒƒƒƒƒƒƒƒ

3 2.9600000 0.1002397 3.3864768 2.8640000 3.0640000

ƒƒƒƒƒƒƒƒƒƒƒƒƒƒƒƒƒƒƒƒƒƒƒƒƒƒƒƒƒƒƒƒƒƒƒƒƒƒƒƒƒƒƒƒƒƒƒƒƒƒƒƒƒƒƒƒƒƒƒƒƒƒƒƒƒƒƒƒƒƒƒƒƒƒƒƒƒƒƒƒƒƒ

------------------------------------------- **trat=10** --------------------------------------------

Analysis Variable : Xmax

Coeff of

N Mean Std Dev Variation Minimum Maximum

ƒƒƒƒƒƒƒƒƒƒƒƒƒƒƒƒƒƒƒƒƒƒƒƒƒƒƒƒƒƒƒƒƒƒƒƒƒƒƒƒƒƒƒƒƒƒƒƒƒƒƒƒƒƒƒƒƒƒƒƒƒƒƒƒƒƒƒƒƒƒƒƒƒƒƒƒƒƒƒƒƒƒ

3 2.1763333 0.2293171 10.5368557 1.9590000 2.4160000

ƒƒƒƒƒƒƒƒƒƒƒƒƒƒƒƒƒƒƒƒƒƒƒƒƒƒƒƒƒƒƒƒƒƒƒƒƒƒƒƒƒƒƒƒƒƒƒƒƒƒƒƒƒƒƒƒƒƒƒƒƒƒƒƒƒƒƒƒƒƒƒƒƒƒƒƒƒƒƒƒƒƒ

------------------------------------------- **trat=25** --------------------------------------------

Analysis Variable : Xmax

Coeff of

N Mean Std Dev Variation Minimum Maximum

ƒƒƒƒƒƒƒƒƒƒƒƒƒƒƒƒƒƒƒƒƒƒƒƒƒƒƒƒƒƒƒƒƒƒƒƒƒƒƒƒƒƒƒƒƒƒƒƒƒƒƒƒƒƒƒƒƒƒƒƒƒƒƒƒƒƒƒƒƒƒƒƒƒƒƒƒƒƒƒƒƒƒ

3 2.1596667 0.0238607 1.1048329 2.1430000 2.1870000

ƒƒƒƒƒƒƒƒƒƒƒƒƒƒƒƒƒƒƒƒƒƒƒƒƒƒƒƒƒƒƒƒƒƒƒƒƒƒƒƒƒƒƒƒƒƒƒƒƒƒƒƒƒƒƒƒƒƒƒƒƒƒƒƒƒƒƒƒƒƒƒƒƒƒƒƒƒƒƒƒƒƒ

The GLM Procedure

Class Level Information

Class Levels Values

trat 4 0 5 10 25

Number of observations 12

The GLM Procedure

**Dependent Variable: Xmax**

Sum of

Source DF Squares Mean Square F Value Pr > F

Model 3 2.61800092 0.87266697 28.68 **0.0001**

Error 8 0.24344200 0.03043025

Corrected Total 11 2.86144292

R-Square Coeff Var Root MSE Xmax Mean

0.914923 7.796611 0.174443 2.237417

Source DF Type I SS Mean Square F Value Pr > F

trat 3 2.61800092 0.87266697 28.68 0.0001

Source DF Type III SS Mean Square F Value Pr > F

trat 3 2.61800092 0.87266697 28.68 0.0001

The GLM Procedure

**Duncan's Multiple Range Test for Xmax**

NOTE: This test controls the Type I comparisonwise error rate. not the experimentwise error

rate.

Alpha 0.05

Error Degrees of Freedom 8

Error Mean Square 0.03043

Number of Means 2 3 4

Critical Range .3284 .3423 .3500

Means with the same letter are not significantly different.

Duncan Grouping Mean N trat

**A 2.9600 3 5**

**B 2.1763 3 10**

**B 2.1597 3 25**

**C 1.6537 3 0**

**Pmax**

General Descriptive Statistics

The MEANS Procedure

Coeff of

Variable N Mean Std Dev Variation Minimum Maximum

ƒƒƒƒƒƒƒƒƒƒƒƒƒƒƒƒƒƒƒƒƒƒƒƒƒƒƒƒƒƒƒƒƒƒƒƒƒƒƒƒƒƒƒƒƒƒƒƒƒƒƒƒƒƒƒƒƒƒƒƒƒƒƒƒƒƒƒƒƒƒƒƒƒƒƒƒƒƒƒƒƒƒƒƒƒƒƒƒƒƒƒƒƒƒ

trat 12 10.0000000 9.7700842 97.7008421 0 25.0000000

pmax 12 0.1407333 0.0343547 24.4112099 0.0956000 0.1875000

ƒƒƒƒƒƒƒƒƒƒƒƒƒƒƒƒƒƒƒƒƒƒƒƒƒƒƒƒƒƒƒƒƒƒƒƒƒƒƒƒƒƒƒƒƒƒƒƒƒƒƒƒƒƒƒƒƒƒƒƒƒƒƒƒƒƒƒƒƒƒƒƒƒƒƒƒƒƒƒƒƒƒƒƒƒƒƒƒƒƒƒƒƒƒ

-------------------------------------------- **trat=0** --------------------------------------------

The MEANS Procedure

Analysis Variable : pmax

Coeff of

N Mean Std Dev Variation Minimum Maximum

ƒƒƒƒƒƒƒƒƒƒƒƒƒƒƒƒƒƒƒƒƒƒƒƒƒƒƒƒƒƒƒƒƒƒƒƒƒƒƒƒƒƒƒƒƒƒƒƒƒƒƒƒƒƒƒƒƒƒƒƒƒƒƒƒƒƒƒƒƒƒƒƒƒƒƒƒƒƒƒƒƒƒ

3 0.1011667 0.0048645 4.8083992 0.0956000 0.1046000

ƒƒƒƒƒƒƒƒƒƒƒƒƒƒƒƒƒƒƒƒƒƒƒƒƒƒƒƒƒƒƒƒƒƒƒƒƒƒƒƒƒƒƒƒƒƒƒƒƒƒƒƒƒƒƒƒƒƒƒƒƒƒƒƒƒƒƒƒƒƒƒƒƒƒƒƒƒƒƒƒƒƒ

-------------------------------------------- **trat=5** --------------------------------------------

Analysis Variable : pmax

Coeff of

N Mean Std Dev Variation Minimum Maximum

ƒƒƒƒƒƒƒƒƒƒƒƒƒƒƒƒƒƒƒƒƒƒƒƒƒƒƒƒƒƒƒƒƒƒƒƒƒƒƒƒƒƒƒƒƒƒƒƒƒƒƒƒƒƒƒƒƒƒƒƒƒƒƒƒƒƒƒƒƒƒƒƒƒƒƒƒƒƒƒƒƒƒ

3 0.1793333 0.0078258 4.3638357 0.1719000 0.1875000

ƒƒƒƒƒƒƒƒƒƒƒƒƒƒƒƒƒƒƒƒƒƒƒƒƒƒƒƒƒƒƒƒƒƒƒƒƒƒƒƒƒƒƒƒƒƒƒƒƒƒƒƒƒƒƒƒƒƒƒƒƒƒƒƒƒƒƒƒƒƒƒƒƒƒƒƒƒƒƒƒƒƒ

------------------------------------------- **trat=10** --------------------------------------------

Analysis Variable : pmax

Coeff of

N Mean Std Dev Variation Minimum Maximum

ƒƒƒƒƒƒƒƒƒƒƒƒƒƒƒƒƒƒƒƒƒƒƒƒƒƒƒƒƒƒƒƒƒƒƒƒƒƒƒƒƒƒƒƒƒƒƒƒƒƒƒƒƒƒƒƒƒƒƒƒƒƒƒƒƒƒƒƒƒƒƒƒƒƒƒƒƒƒƒƒƒƒ

3 0.1628667 0.0203493 12.4944433 0.1488000 0.1862000

ƒƒƒƒƒƒƒƒƒƒƒƒƒƒƒƒƒƒƒƒƒƒƒƒƒƒƒƒƒƒƒƒƒƒƒƒƒƒƒƒƒƒƒƒƒƒƒƒƒƒƒƒƒƒƒƒƒƒƒƒƒƒƒƒƒƒƒƒƒƒƒƒƒƒƒƒƒƒƒƒƒƒ

------------------------------------------- **trat=2.5** --------------------------------------------

Analysis Variable : pmax

Coeff of

N Mean Std Dev Variation Minimum Maximum

ƒƒƒƒƒƒƒƒƒƒƒƒƒƒƒƒƒƒƒƒƒƒƒƒƒƒƒƒƒƒƒƒƒƒƒƒƒƒƒƒƒƒƒƒƒƒƒƒƒƒƒƒƒƒƒƒƒƒƒƒƒƒƒƒƒƒƒƒƒƒƒƒƒƒƒƒƒƒƒƒƒƒ

3 0.1195667 0.0015044 1.2582419 0.1186000 0.1213000

ƒƒƒƒƒƒƒƒƒƒƒƒƒƒƒƒƒƒƒƒƒƒƒƒƒƒƒƒƒƒƒƒƒƒƒƒƒƒƒƒƒƒƒƒƒƒƒƒƒƒƒƒƒƒƒƒƒƒƒƒƒƒƒƒƒƒƒƒƒƒƒƒƒƒƒƒƒƒƒƒƒƒ

The GLM Procedure

Class Level Information

Class Levels Values

trat 4 0 5 10 25

Number of observations 12

The SAS System 08:12 Monday. September 6. 2019 4

The GLM Procedure

**Dependent Variable: pmax**

Sum of

Source DF Squares Mean Square F Value Pr > F

Model 3 0.01198018 0.00399339 31.87 **<.0001**

Error 8 0.00100253 0.00012532

Corrected Total 11 0.01298271

R-Square Coeff Var Root MSE pmax Mean

0.922780 7.954374 0.011194 0.140733

Source DF Type I SS Mean Square F Value Pr > F

trat 3 0.01198018 0.00399339 31.87 <.0001

Source DF Type III SS Mean Square F Value Pr > F

trat 3 0.01198018 0.00399339 31.87 <.0001

The GLM Procedure

**Duncan's Multiple Range Test for pmax**

NOTE: This test controls the Type I comparisonwise error rate. not the experimentwise error

rate.

Alpha 0.05

Error Degrees of Freedom 8

Error Mean Square 0.000125

Number of Means 2 3 4

Critical Range .02108 .02196 .02246

Means with the same letter are not significantly different.

Duncan Grouping Mean N trat

**A 0.179333 3 5**

**A 0.162867 3 10**

**B 0.119567 3 25**

**B 0.101167 3 0**

**µ**

General Descriptive Statistics

The MEANS Procedure

Coeff of

Variable N Mean Std Dev Variation Minimum Maximum

ƒƒƒƒƒƒƒƒƒƒƒƒƒƒƒƒƒƒƒƒƒƒƒƒƒƒƒƒƒƒƒƒƒƒƒƒƒƒƒƒƒƒƒƒƒƒƒƒƒƒƒƒƒƒƒƒƒƒƒƒƒƒƒƒƒƒƒƒƒƒƒƒƒƒƒƒƒƒƒƒƒƒƒƒƒƒƒƒƒƒƒƒƒƒ

trat 12 10.0000000 9.7700842 97.7008421 0 25.0000000

mu 12 0.8782500 0.7851424 89.3985069 0.1750000 2.1020000

ƒƒƒƒƒƒƒƒƒƒƒƒƒƒƒƒƒƒƒƒƒƒƒƒƒƒƒƒƒƒƒƒƒƒƒƒƒƒƒƒƒƒƒƒƒƒƒƒƒƒƒƒƒƒƒƒƒƒƒƒƒƒƒƒƒƒƒƒƒƒƒƒƒƒƒƒƒƒƒƒƒƒƒƒƒƒƒƒƒƒƒƒƒƒ

-------------------------------------------- **trat=0** --------------------------------------------

The MEANS Procedure

Analysis Variable : mu

Coeff of

N Mean Std Dev Variation Minimum Maximum

ƒƒƒƒƒƒƒƒƒƒƒƒƒƒƒƒƒƒƒƒƒƒƒƒƒƒƒƒƒƒƒƒƒƒƒƒƒƒƒƒƒƒƒƒƒƒƒƒƒƒƒƒƒƒƒƒƒƒƒƒƒƒƒƒƒƒƒƒƒƒƒƒƒƒƒƒƒƒƒƒƒƒ

3 0.1830000 0.0105830 5.7830630 0.1750000 0.1950000

ƒƒƒƒƒƒƒƒƒƒƒƒƒƒƒƒƒƒƒƒƒƒƒƒƒƒƒƒƒƒƒƒƒƒƒƒƒƒƒƒƒƒƒƒƒƒƒƒƒƒƒƒƒƒƒƒƒƒƒƒƒƒƒƒƒƒƒƒƒƒƒƒƒƒƒƒƒƒƒƒƒƒ

-------------------------------------------- **trat=5** --------------------------------------------

Analysis Variable : mu

Coeff of

N Mean Std Dev Variation Minimum Maximum

ƒƒƒƒƒƒƒƒƒƒƒƒƒƒƒƒƒƒƒƒƒƒƒƒƒƒƒƒƒƒƒƒƒƒƒƒƒƒƒƒƒƒƒƒƒƒƒƒƒƒƒƒƒƒƒƒƒƒƒƒƒƒƒƒƒƒƒƒƒƒƒƒƒƒƒƒƒƒƒƒƒƒ

3 1.0240000 0.0258650 2.5258823 1.0010000 1.0520000

ƒƒƒƒƒƒƒƒƒƒƒƒƒƒƒƒƒƒƒƒƒƒƒƒƒƒƒƒƒƒƒƒƒƒƒƒƒƒƒƒƒƒƒƒƒƒƒƒƒƒƒƒƒƒƒƒƒƒƒƒƒƒƒƒƒƒƒƒƒƒƒƒƒƒƒƒƒƒƒƒƒƒ

------------------------------------------- **trat=10** --------------------------------------------

Analysis Variable : mu

Coeff of

N Mean Std Dev Variation Minimum Maximum

ƒƒƒƒƒƒƒƒƒƒƒƒƒƒƒƒƒƒƒƒƒƒƒƒƒƒƒƒƒƒƒƒƒƒƒƒƒƒƒƒƒƒƒƒƒƒƒƒƒƒƒƒƒƒƒƒƒƒƒƒƒƒƒƒƒƒƒƒƒƒƒƒƒƒƒƒƒƒƒƒƒƒ

3 2.0480000 0.0540000 2.6367187 1.9940000 2.1020000

ƒƒƒƒƒƒƒƒƒƒƒƒƒƒƒƒƒƒƒƒƒƒƒƒƒƒƒƒƒƒƒƒƒƒƒƒƒƒƒƒƒƒƒƒƒƒƒƒƒƒƒƒƒƒƒƒƒƒƒƒƒƒƒƒƒƒƒƒƒƒƒƒƒƒƒƒƒƒƒƒƒƒ

------------------------------------------- **trat=25** --------------------------------------------

Analysis Variable : mu

Coeff of

N Mean Std Dev Variation Minimum Maximum

ƒƒƒƒƒƒƒƒƒƒƒƒƒƒƒƒƒƒƒƒƒƒƒƒƒƒƒƒƒƒƒƒƒƒƒƒƒƒƒƒƒƒƒƒƒƒƒƒƒƒƒƒƒƒƒƒƒƒƒƒƒƒƒƒƒƒƒƒƒƒƒƒƒƒƒƒƒƒƒƒƒƒ

3 0.2580000 0.0175784 6.8133317 0.2450000 0.2780000

ƒƒƒƒƒƒƒƒƒƒƒƒƒƒƒƒƒƒƒƒƒƒƒƒƒƒƒƒƒƒƒƒƒƒƒƒƒƒƒƒƒƒƒƒƒƒƒƒƒƒƒƒƒƒƒƒƒƒƒƒƒƒƒƒƒƒƒƒƒƒƒƒƒƒƒƒƒƒƒƒƒƒ

The GLM Procedure

Class Level Information

Class Levels Values

trat 4 0 5 10 25

Number of observations 12

The GLM Procedure

**Dependent Variable: mu**

Sum of

Source DF Squares Mean Square F Value Pr > F

Model 3 6.77292225 2.25764075 2254.26 **<.0001**

Error 8 0.00801200 0.00100150

Corrected Total 11 6.78093425

R-Square Coeff Var Root MSE mu Mean

0.998818 3.603357 0.031646 0.878250

Source DF Type I SS Mean Square F Value Pr > F

trat 3 6.77292225 2.25764075 2254.26 <.0001

Source DF Type III SS Mean Square F Value Pr > F

trat 3 6.77292225 2.25764075 2254.26 <.0001

The GLM Procedure

**Duncan's Multiple Range Test for mu**

NOTE: This test controls the Type I comparisonwise error rate. not the experimentwise error

rate.

Alpha 0.05

Error Degrees of Freedom 8

Error Mean Square 0.001001

Number of Means 2 3 4

Critical Range .05958 .06209 .06350

Means with the same letter are not significantly different.

Duncan Grouping Mean N trat

**A 2.04800 3 10**

**B 1.02400 3 5**

**C 0.25800 3 25**

**D 0.18300 3 0**

**Biochemical composition**

**General Descriptive Statistics** The MEANS Procedure

Coeff of

Variable N Mean Std Dev Variation Minimum Maximum

ƒƒƒƒƒƒƒƒƒƒƒƒƒƒƒƒƒƒƒƒƒƒƒƒƒƒƒƒƒƒƒƒƒƒƒƒƒƒƒƒƒƒƒƒƒƒƒƒƒƒƒƒƒƒƒƒƒƒƒƒƒƒƒƒƒƒƒƒƒƒƒƒƒƒƒƒƒƒƒƒƒƒƒƒƒƒƒƒƒƒƒƒƒƒ

trat 12 4.3750000 3.8619649 88.2734830 0 10.0000000

rep 12 2.0000000 0.8528029 42.6401433 1.0000000 3.0000000

dry 12 92.7258333 1.9519848 2.1051143 90.2100000 96.0100000

pt 12 52.5875000 11.2960251 21.4804376 39.4100000 67.7100000

fat 12 3.2966667 1.2635976 38.3295518 2.0000000 5.6400000

CARBO 12 34.7325000 10.5381326 30.3408410 22.0400000 48.7000000

ash 12 10.1600000 4.1440319 40.7877151 5.7500000 14.2400000

ƒƒƒƒƒƒƒƒƒƒƒƒƒƒƒƒƒƒƒƒƒƒƒƒƒƒƒƒƒƒƒƒƒƒƒƒƒƒƒƒƒƒƒƒƒƒƒƒƒƒƒƒƒƒƒƒƒƒƒƒƒƒƒƒƒƒƒƒƒƒƒƒƒƒƒƒƒƒƒƒƒƒƒƒƒƒƒƒƒƒƒƒƒƒ

--------------------------------------------- trat=0 ----------------------------------------------

Coeff of

Variable N Mean Std Dev Variation Minimum Maximum

ƒƒƒƒƒƒƒƒƒƒƒƒƒƒƒƒƒƒƒƒƒƒƒƒƒƒƒƒƒƒƒƒƒƒƒƒƒƒƒƒƒƒƒƒƒƒƒƒƒƒƒƒƒƒƒƒƒƒƒƒƒƒƒƒƒƒƒƒƒƒƒƒƒƒƒƒƒƒƒƒƒƒƒƒƒƒƒƒƒƒƒƒƒƒ

dry 3 93.6500000 1.5974667 1.7057840 92.4800000 95.4700000

pt 3 65.5466667 1.8754288 2.8612116 64.3800000 67.7100000

fat 3 5.1800000 0.4132796 7.9783700 4.8400000 5.6400000

CARBO 3 27.1700000 1.5733086 5.7906096 25.3600000 28.2100000

ash 3 6.0366667 0.3817504 6.3238601 5.7500000 6.4700000

ƒƒƒƒƒƒƒƒƒƒƒƒƒƒƒƒƒƒƒƒƒƒƒƒƒƒƒƒƒƒƒƒƒƒƒƒƒƒƒƒƒƒƒƒƒƒƒƒƒƒƒƒƒƒƒƒƒƒƒƒƒƒƒƒƒƒƒƒƒƒƒƒƒƒƒƒƒƒƒƒƒƒƒƒƒƒƒƒƒƒƒƒƒƒ

-------------------------------------------- trat=2.5 ---------------------------------------------

Coeff of

Variable N Mean Std Dev Variation Minimum Maximum

ƒƒƒƒƒƒƒƒƒƒƒƒƒƒƒƒƒƒƒƒƒƒƒƒƒƒƒƒƒƒƒƒƒƒƒƒƒƒƒƒƒƒƒƒƒƒƒƒƒƒƒƒƒƒƒƒƒƒƒƒƒƒƒƒƒƒƒƒƒƒƒƒƒƒƒƒƒƒƒƒƒƒƒƒƒƒƒƒƒƒƒƒƒƒ

dry 3 91.7666667 0.5762233 0.6279223 91.1700000 92.3200000

pt 3 60.6200000 0.3019934 0.4981745 60.3400000 60.9400000

fat 3 2.0433333 0.0378594 1.8528249 2.0000000 2.0700000

CARBO 3 23.2866667 1.5413090 6.6188476 22.0400000 25.0100000

ash 3 14.2100000 0.0300000 0.2111189 14.1800000 14.2400000

ƒƒƒƒƒƒƒƒƒƒƒƒƒƒƒƒƒƒƒƒƒƒƒƒƒƒƒƒƒƒƒƒƒƒƒƒƒƒƒƒƒƒƒƒƒƒƒƒƒƒƒƒƒƒƒƒƒƒƒƒƒƒƒƒƒƒƒƒƒƒƒƒƒƒƒƒƒƒƒƒƒƒƒƒƒƒƒƒƒƒƒƒƒƒ

--------------------------------------------- trat=5 ----------------------------------------------

Coeff of

Variable N Mean Std Dev Variation Minimum Maximum

ƒƒƒƒƒƒƒƒƒƒƒƒƒƒƒƒƒƒƒƒƒƒƒƒƒƒƒƒƒƒƒƒƒƒƒƒƒƒƒƒƒƒƒƒƒƒƒƒƒƒƒƒƒƒƒƒƒƒƒƒƒƒƒƒƒƒƒƒƒƒƒƒƒƒƒƒƒƒƒƒƒƒƒƒƒƒƒƒƒƒƒƒƒƒ

dry 3 90.5700000 0.5150728 0.5687013 90.2100000 91.1600000

pt 3 44.5633333 0.7441326 1.6698316 43.7500000 45.2100000

fat 3 2.5600000 0.2100000 8.2031250 2.3200000 2.7100000

CARBO 3 47.8266667 0.7849416 1.6412217 47.1800000 48.7000000

ash 3 14.0333333 0.1446836 1.0309993 13.9400000 14.2000000

ƒƒƒƒƒƒƒƒƒƒƒƒƒƒƒƒƒƒƒƒƒƒƒƒƒƒƒƒƒƒƒƒƒƒƒƒƒƒƒƒƒƒƒƒƒƒƒƒƒƒƒƒƒƒƒƒƒƒƒƒƒƒƒƒƒƒƒƒƒƒƒƒƒƒƒƒƒƒƒƒƒƒƒƒƒƒƒƒƒƒƒƒƒƒ

--------------------------------------------- trat=10 ---------------------------------------------

Coeff of

Variable N Mean Std Dev Variation Minimum Maximum

ƒƒƒƒƒƒƒƒƒƒƒƒƒƒƒƒƒƒƒƒƒƒƒƒƒƒƒƒƒƒƒƒƒƒƒƒƒƒƒƒƒƒƒƒƒƒƒƒƒƒƒƒƒƒƒƒƒƒƒƒƒƒƒƒƒƒƒƒƒƒƒƒƒƒƒƒƒƒƒƒƒƒƒƒƒƒƒƒƒƒƒƒƒƒ

dry 3 94.9166667 0.9873871 1.0402674 94.0900000 96.0100000

pt 3 39.6200000 0.3637307 0.9180481 39.4100000 40.0400000

fat 3 3.4033333 0.2400694 7.0539501 3.2500000 3.6800000

CARBO 3 40.6466667 3.6565877 8.9960333 36.8700000 44.1700000

ash 3 6.3600000 0.1153256 1.8132960 6.2300000 6.4500000

ƒƒƒƒƒƒƒƒƒƒƒƒƒƒƒƒƒƒƒƒƒƒƒƒƒƒƒƒƒƒƒƒƒƒƒƒƒƒƒƒƒƒƒƒƒƒƒƒƒƒƒƒƒƒƒƒƒƒƒƒƒƒƒƒƒƒƒƒƒƒƒƒƒƒƒƒƒƒƒƒƒƒƒƒƒƒƒƒƒƒƒƒƒƒ

The GLM Procedure

Class Level Information

Class Levels Values

trat 4 0 2.5 5 10

Number of observations 12

**Dependent Variable: dry**

Sum of

Source DF Squares Mean Square F Value Pr > F

Model 3 33.66435833 11.22145278 10.88 **0.0034**

Error 8 8.24833333 1.03104167

Corrected Total 11 41.91269167

R-Square Coeff Var Root MSE dry Mean

0.803202 1.095059 1.015402 92.72583

Source DF Type I SS Mean Square F Value Pr > F

trat 3 33.66435833 11.22145278 10.88 0.0034

Source DF Type III SS Mean Square F Value Pr > F

trat 3 33.66435833 11.22145278 10.88 0.0034

**Dependent Variable: pt**

Sum of

Source DF Squares Mean Square F Value Pr > F

Model 3 1395.013092 465.004364 433.12 **<.0001**

Error 8 8.588933 1.073617

Corrected Total 11 1403.602025

R-Square Coeff Var Root MSE pt Mean

0.993881 1.970344 1.036155 52.58750

Source DF Type I SS Mean Square F Value Pr > F

trat 3 1395.013092 465.004364 433.12 <.0001

Source DF Type III SS Mean Square F Value Pr > F

trat 3 1395.013092 465.004364 433.12 <.0001

**Dependent Variable: fat**

Sum of

Source DF Squares Mean Square F Value Pr > F

Model 3 17.01553333 5.67184444 82.81 **<.0001**

Error 8 0.54793333 0.06849167

Corrected Total 11 17.56346667

R-Square Coeff Var Root MSE fat Mean

0.968803 7.938598 0.261709 3.296667

Source DF Type I SS Mean Square F Value Pr > F

trat 3 17.01553333 5.67184444 82.81 <.0001

Source DF Type III SS Mean Square F Value Pr > F

trat 3 17.01553333 5.67184444 82.81 <.0001

**Dependent Variable: CARBO**

Sum of

Source DF Squares Mean Square F Value Pr > F

Model 3 1183.899225 394.633075 83.80 **<.0001**

Error 8 37.675400 4.709425

Corrected Total 11 1221.574625

R-Square Coeff Var Root MSE CARBO Mean

0.969158 6.248099 2.170121 34.73250

Source DF Type I SS Mean Square F Value Pr > F

trat 3 1183.899225 394.633075 83.80 <.0001

Source DF Type III SS Mean Square F Value Pr > F

trat 3 1183.899225 394.633075 83.80 <.0001

**Dependent Variable: ash**

Sum of

Source DF Squares Mean Square F Value Pr > F

Model 3 188.5412667 62.8470889 1389.91 **<.0001**

Error 8 0.3617333 0.0452167

Corrected Total 11 188.9030000

R-Square Coeff Var Root MSE ash Mean

0.998085 2.092934 0.212642 10.16000

Source DF Type I SS Mean Square F Value Pr > F

trat 3 188.5412667 62.8470889 1389.91 <.0001

Source DF Type III SS Mean Square F Value Pr > F

trat 3 188.5412667 62.8470889 1389.91 <.0001

**Teste de Duncan**

The GLM Procedure

**Duncan's Multiple Range Test for dry**

NOTE: This test controls the Type I comparisonwise error rate. not the experimentwise error rate.

Alpha 0.05

Error Degrees of Freedom 8

Error Mean Square 1.031042

Number of Means 2 3 4

Critical Range 1.912 1.992 2.037

Means with the same letter are not significantly different.

Duncan Grouping Mean N trat

**A 94.9167 3 10**

**B A 93.6500 3 0**

**B C 91.7667 3 2.5**

**C 90.5700 3 5**

**Duncan's Multiple Range Test for pt**

NOTE: This test controls the Type I comparisonwise error rate. not the experimentwise error rate.

Alpha 0.05

Error Degrees of Freedom 8

Error Mean Square 1.073617

Number of Means 2 3 4

Critical Range 1.951 2.033 2.079

Means with the same letter are not significantly different.

Duncan Grouping Mean N trat

**A 65.5467 3 0**

**B 60.6200 3 2.5**

**C 44.5633 3 5**

**D 39.6200 3 10**

**Duncan's Multiple Range Test for fat**

NOTE: This test controls the Type I comparisonwise error rate. not the experimentwise error rate.

Alpha 0.05

Error Degrees of Freedom 8

Error Mean Square 0.068492

Number of Means 2 3 4

Critical Range .4928 .5135 .5251

Means with the same letter are not significantly different.

Duncan Grouping Mean N trat

**A 5.1800 3 0**

**B 3.4033 3 10**

**C 2.5600 3 5**

**D 2.0433 3 2.5**

**Duncan's Multiple Range Test for CARBOHYDRATE**

NOTE: This test controls the Type I comparisonwise error rate. not the experimentwise error rate.

Alpha 0.05

Error Degrees of Freedom 8

Error Mean Square 4.709425

Number of Means 2 3 4

Critical Range 4.086 4.258 4.354

Means with the same letter are not significantly different.

Duncan Grouping Mean N trat

**A 47.827 3 5**

**B 40.647 3 10**

**C 27.170 3 0**

**C 23.287 3 2.5**

**Duncan's Multiple Range Test for ash**

NOTE: This test controls the Type I comparisonwise error rate. not the experimentwise error rate.

Alpha 0.05

Error Degrees of Freedom 8

Error Mean Square 0.045217

Number of Means 2 3 4

Critical Range .4004 .4172 .4266

Means with the same letter are not significantly different.

Duncan Grouping Mean N trat

**A 14.2100 3 2.5**

**A 14.0333 3 5**

**B 6.3600 3 10**

**B 6.0367 3 0**

**Phenolics compounds**

**General Descriptive Statistics**

Coeff of

Variable N Mean Std Dev Variation Minimum Maximum

ƒƒƒƒƒƒƒƒƒƒƒƒƒƒƒƒƒƒƒƒƒƒƒƒƒƒƒƒƒƒƒƒƒƒƒƒƒƒƒƒƒƒƒƒƒƒƒƒƒƒƒƒƒƒƒƒƒƒƒƒƒƒƒƒƒƒƒƒƒƒƒƒƒƒƒƒƒƒƒƒƒƒƒƒƒƒƒƒƒƒƒƒƒƒ

whey 12 4.3750000 3.8619649 88.2734830 0 10.0000000

phenol 12 29.8500000 4.6672262 15.6355985 22.2200000 33.9000000

ƒƒƒƒƒƒƒƒƒƒƒƒƒƒƒƒƒƒƒƒƒƒƒƒƒƒƒƒƒƒƒƒƒƒƒƒƒƒƒƒƒƒƒƒƒƒƒƒƒƒƒƒƒƒƒƒƒƒƒƒƒƒƒƒƒƒƒƒƒƒƒƒƒƒƒƒƒƒƒƒƒƒƒƒƒƒƒƒƒƒƒƒƒƒ

**Estatística Descritiva por nível de Whey**

-------------------------------------------- whey=0 --------------------------------------------

Analysis Variable : phenol

Coeff of

N Mean Std Dev Variation Minimum Maximum

ƒƒƒƒƒƒƒƒƒƒƒƒƒƒƒƒƒƒƒƒƒƒƒƒƒƒƒƒƒƒƒƒƒƒƒƒƒƒƒƒƒƒƒƒƒƒƒƒƒƒƒƒƒƒƒƒƒƒƒƒƒƒƒƒƒƒƒƒƒƒƒƒƒƒƒƒƒƒƒƒƒƒ

3 33.1966667 0.9166424 2.7612484 32.1600000 33.9000000

ƒƒƒƒƒƒƒƒƒƒƒƒƒƒƒƒƒƒƒƒƒƒƒƒƒƒƒƒƒƒƒƒƒƒƒƒƒƒƒƒƒƒƒƒƒƒƒƒƒƒƒƒƒƒƒƒƒƒƒƒƒƒƒƒƒƒƒƒƒƒƒƒƒƒƒƒƒƒƒƒƒƒ

------------------------------------------- whey=2.5 -------------------------------------------

Coeff of

N Mean Std Dev Variation Minimum Maximum

ƒƒƒƒƒƒƒƒƒƒƒƒƒƒƒƒƒƒƒƒƒƒƒƒƒƒƒƒƒƒƒƒƒƒƒƒƒƒƒƒƒƒƒƒƒƒƒƒƒƒƒƒƒƒƒƒƒƒƒƒƒƒƒƒƒƒƒƒƒƒƒƒƒƒƒƒƒƒƒƒƒƒ

3 30.6000000 0.4330127 1.4150742 30.1000000 30.8500000

ƒƒƒƒƒƒƒƒƒƒƒƒƒƒƒƒƒƒƒƒƒƒƒƒƒƒƒƒƒƒƒƒƒƒƒƒƒƒƒƒƒƒƒƒƒƒƒƒƒƒƒƒƒƒƒƒƒƒƒƒƒƒƒƒƒƒƒƒƒƒƒƒƒƒƒƒƒƒƒƒƒƒ

-------------------------------------------- whey=5 --------------------------------------------

Coeff of

N Mean Std Dev Variation Minimum Maximum

ƒƒƒƒƒƒƒƒƒƒƒƒƒƒƒƒƒƒƒƒƒƒƒƒƒƒƒƒƒƒƒƒƒƒƒƒƒƒƒƒƒƒƒƒƒƒƒƒƒƒƒƒƒƒƒƒƒƒƒƒƒƒƒƒƒƒƒƒƒƒƒƒƒƒƒƒƒƒƒƒƒƒ

3 33.2300000 0.3551056 1.0686296 32.8700000 33.5800000

ƒƒƒƒƒƒƒƒƒƒƒƒƒƒƒƒƒƒƒƒƒƒƒƒƒƒƒƒƒƒƒƒƒƒƒƒƒƒƒƒƒƒƒƒƒƒƒƒƒƒƒƒƒƒƒƒƒƒƒƒƒƒƒƒƒƒƒƒƒƒƒƒƒƒƒƒƒƒƒƒƒƒ

------------------------------------------- whey=10 --------------------------------------------

Coeff of

N Mean Std Dev Variation Minimum Maximum

ƒƒƒƒƒƒƒƒƒƒƒƒƒƒƒƒƒƒƒƒƒƒƒƒƒƒƒƒƒƒƒƒƒƒƒƒƒƒƒƒƒƒƒƒƒƒƒƒƒƒƒƒƒƒƒƒƒƒƒƒƒƒƒƒƒƒƒƒƒƒƒƒƒƒƒƒƒƒƒƒƒƒ

3 22.3733333 0.1457166 0.6512960 22.2200000 22.5100000

ƒƒƒƒƒƒƒƒƒƒƒƒƒƒƒƒƒƒƒƒƒƒƒƒƒƒƒƒƒƒƒƒƒƒƒƒƒƒƒƒƒƒƒƒƒƒƒƒƒƒƒƒƒƒƒƒƒƒƒƒƒƒƒƒƒƒƒƒƒƒƒƒƒƒƒƒƒƒƒƒƒƒ

The GLM Procedure

Class Level Information

Class Levels Values

whey 4 0 2.5 5 10

Number of observations 12

**Dependent Variable: phenol**

Sum of

Source DF Squares Mean Square F Value Pr > F

Model 3 237.2628667 79.0876222 269.22 **<.0001**

Error 8 2.3501333 0.2937667

Corrected Total 11 239.6130000

R-Square Coeff Var Root MSE phenol Mean

0.990192 1.815754 0.542002 29.85000

Source DF Type I SS Mean Square F Value Pr > F

whey 3 237.2628667 79.0876222 269.22 <.0001

Source DF Type III SS Mean Square F Value Pr > F

whey 3 237.2628667 79.0876222 269.22 <.0001

**Duncan's Multiple Range Test for phenol**

NOTE: This test controls the Type I comparisonwise error rate. not the experimentwise error

rate.

Alpha 0.05

Error Degrees of Freedom 8

Error Mean Square 0.293767

Number of Means 2 3 4

Critical Range 1.020 1.063 1.087

Means with the same letter are not significantly different.

**Duncan Grouping Mean N whey**

**A 33.2300 3 5**

**A 33.1967 3 0**

**B 30.6000 3 2.5**

**C 22.3733 3 10**

**Antioxidant capacity**

**General Descriptive Statistics**

The MEANS Procedure

Coeff of

Variable N Mean Std Dev Variation Minimum Maximum

ƒƒƒƒƒƒƒƒƒƒƒƒƒƒƒƒƒƒƒƒƒƒƒƒƒƒƒƒƒƒƒƒƒƒƒƒƒƒƒƒƒƒƒƒƒƒƒƒƒƒƒƒƒƒƒƒƒƒƒƒƒƒƒƒƒƒƒƒƒƒƒƒƒƒƒƒƒƒƒƒƒƒƒƒƒƒƒƒƒƒƒ

Whey 12 4.3750000 3.8619649 88.2734830 0 10.0000000

Replication 12 2.0000000 0.8528029 42.6401433 1.0000000 3.0000000

FRAPsfg 12 20.1316667 9.1930614 45.6646814 14.4000000 36.2300000

FRAPTxg 12 11.7983333 5.3640247 45.4642576 8.4500000 21.1900000

ABTSTxg 12 14.7050000 2.2345530 15.1958724 11.5700000 18.1000000

ƒƒƒƒƒƒƒƒƒƒƒƒƒƒƒƒƒƒƒƒƒƒƒƒƒƒƒƒƒƒƒƒƒƒƒƒƒƒƒƒƒƒƒƒƒƒƒƒƒƒƒƒƒƒƒƒƒƒƒƒƒƒƒƒƒƒƒƒƒƒƒƒƒƒƒƒƒƒƒƒƒƒƒƒƒƒƒƒƒƒƒ

-------------------------------------------- Whey=0 --------------------------------------------

The MEANS Procedure

Coeff of

Variable N Mean Std Dev Variation Minimum Maximum

ƒƒƒƒƒƒƒƒƒƒƒƒƒƒƒƒƒƒƒƒƒƒƒƒƒƒƒƒƒƒƒƒƒƒƒƒƒƒƒƒƒƒƒƒƒƒƒƒƒƒƒƒƒƒƒƒƒƒƒƒƒƒƒƒƒƒƒƒƒƒƒƒƒƒƒƒƒƒƒƒƒƒƒƒƒƒƒƒƒƒƒ

FRAPsfg 3 35.3533333 0.8750048 2.4750276 34.4800000 36.2300000

FRAPTxg 3 20.6800000 0.5100000 2.4661509 20.1700000 21.1900000

ABTSTxg 3 17.6733333 0.5229085 2.9587431 17.0900000 18.1000000

ƒƒƒƒƒƒƒƒƒƒƒƒƒƒƒƒƒƒƒƒƒƒƒƒƒƒƒƒƒƒƒƒƒƒƒƒƒƒƒƒƒƒƒƒƒƒƒƒƒƒƒƒƒƒƒƒƒƒƒƒƒƒƒƒƒƒƒƒƒƒƒƒƒƒƒƒƒƒƒƒƒƒƒƒƒƒƒƒƒƒƒ

------------------------------------------- Whey=2.5 -------------------------------------------

Coeff of

Variable N Mean Std Dev Variation Minimum Maximum

ƒƒƒƒƒƒƒƒƒƒƒƒƒƒƒƒƒƒƒƒƒƒƒƒƒƒƒƒƒƒƒƒƒƒƒƒƒƒƒƒƒƒƒƒƒƒƒƒƒƒƒƒƒƒƒƒƒƒƒƒƒƒƒƒƒƒƒƒƒƒƒƒƒƒƒƒƒƒƒƒƒƒƒƒƒƒƒƒƒƒƒ

FRAPsfg 3 15.2466667 0.0750555 0.4922750 15.1700000 15.3200000

FRAPTxg 3 8.9466667 0.0450925 0.5040145 8.9000000 8.9900000

ABTSTxg 3 14.8433333 0.1401190 0.9439861 14.7300000 15.0000000

ƒƒƒƒƒƒƒƒƒƒƒƒƒƒƒƒƒƒƒƒƒƒƒƒƒƒƒƒƒƒƒƒƒƒƒƒƒƒƒƒƒƒƒƒƒƒƒƒƒƒƒƒƒƒƒƒƒƒƒƒƒƒƒƒƒƒƒƒƒƒƒƒƒƒƒƒƒƒƒƒƒƒƒƒƒƒƒƒƒƒƒ

-------------------------------------------- Whey=5 --------------------------------------------

Coeff of

Variable N Mean Std Dev Variation Minimum Maximum

ƒƒƒƒƒƒƒƒƒƒƒƒƒƒƒƒƒƒƒƒƒƒƒƒƒƒƒƒƒƒƒƒƒƒƒƒƒƒƒƒƒƒƒƒƒƒƒƒƒƒƒƒƒƒƒƒƒƒƒƒƒƒƒƒƒƒƒƒƒƒƒƒƒƒƒƒƒƒƒƒƒƒƒƒƒƒƒƒƒƒƒ

FRAPsfg 3 15.3866667 0.1050397 0.6826669 15.2800000 15.4900000

FRAPTxg 3 9.0300000 0.0600000 0.6644518 8.9700000 9.0900000

ABTSTxg 3 14.6200000 0.4453089 3.0458884 14.1300000 15.0000000

ƒƒƒƒƒƒƒƒƒƒƒƒƒƒƒƒƒƒƒƒƒƒƒƒƒƒƒƒƒƒƒƒƒƒƒƒƒƒƒƒƒƒƒƒƒƒƒƒƒƒƒƒƒƒƒƒƒƒƒƒƒƒƒƒƒƒƒƒƒƒƒƒƒƒƒƒƒƒƒƒƒƒƒƒƒƒƒƒƒƒƒ

------------------------------------------- Whey=10 --------------------------------------------

Coeff of

Variable N Mean Std Dev Variation Minimum Maximum

ƒƒƒƒƒƒƒƒƒƒƒƒƒƒƒƒƒƒƒƒƒƒƒƒƒƒƒƒƒƒƒƒƒƒƒƒƒƒƒƒƒƒƒƒƒƒƒƒƒƒƒƒƒƒƒƒƒƒƒƒƒƒƒƒƒƒƒƒƒƒƒƒƒƒƒƒƒƒƒƒƒƒƒƒƒƒƒƒƒƒƒ

FRAPsfg 3 14.5400000 0.1400000 0.9628611 14.4000000 14.6800000

FRAPTxg 3 8.5366667 0.0850490 0.9962789 8.4500000 8.6200000

ABTSTxg 3 11.6833333 0.1401190 1.1993067 11.5700000 11.8400000

ƒƒƒƒƒƒƒƒƒƒƒƒƒƒƒƒƒƒƒƒƒƒƒƒƒƒƒƒƒƒƒƒƒƒƒƒƒƒƒƒƒƒƒƒƒƒƒƒƒƒƒƒƒƒƒƒƒƒƒƒƒƒƒƒƒƒƒƒƒƒƒƒƒƒƒƒƒƒƒƒƒƒƒƒƒƒƒƒƒƒƒ

The GLM Procedure

Class Level Information

Class Levels Values

Whey 4 0 2.5 5 10

Number of observations 12

The GLM Procedure

**Dependent Variable: FRAPsfg**

Sum of

Source DF Squares Mean Square F Value Pr > F

Model 3 928.0323667 309.3441222 1543.06 **<.0001**

Error 8 1.6038000 0.2004750

Corrected Total 11 929.6361667

R-Square Coeff Var Root MSE FRAPsfg Mean

0.998275 2.224080 0.447744 20.13167

Source DF Type I SS Mean Square F Value Pr > F

Whey 3 928.0323667 309.3441222 1543.06 <.0001

Source DF Type III SS Mean Square F Value Pr > F

Whey 3 928.0323667 309.3441222 1543.06 <.0001

**Dependent Variable: FRAPTxg**

Sum of

Source DF Squares Mean Square F Value Pr > F

Model 3 315.9544333 105.3181444 1543.31 **<.0001**

Error 8 0.5459333 0.0682417

Corrected Total 11 316.5003667

R-Square Coeff Var Root MSE FRAPTxg Mean

0.998275 2.214135 0.261231 11.79833

Source DF Type I SS Mean Square F Value Pr > F

Whey 3 315.9544333 105.3181444 1543.31 <.0001

Source DF Type III SS Mean Square F Value Pr > F

Whey 3 315.9544333 105.3181444 1543.31 <.0001

**Dependent Variable: ABTSTxg**

Sum of

Source DF Squares Mean Square F Value Pr > F

Model 3 53.90350000 17.96783333 140.65  **<.0001**

Error 8 1.02200000 0.12775000

Corrected Total 11 54.92550000

R-Square Coeff Var Root MSE ABTSTxg Mean

0.981393 2.430611 0.357421 14.70500

Source DF Type I SS Mean Square F Value Pr > F

Whey 3 53.90350000 17.96783333 140.65 <.0001

Source DF Type III SS Mean Square F Value Pr > F

Whey 3 53.90350000 17.96783333 140.65 <.0001

**Duncan's Multiple Range Test for FRAPsfg**

NOTE: This test controls the Type I comparisonwise error rate. not the experimentwise error

rate.

Alpha 0.05

Error Degrees of Freedom 8

Error Mean Square 0.200475

Number of Means 2 3 4

Critical Range .8430 .8785 .8984

Means with the same letter are not significantly different.

**Duncan Grouping Mean N Whey**

**A 35.3533 3 0**

**B 15.3867 3 5**

**B 15.2467 3 2.5**

**B 14.5400 3 10**

**Duncan's Multiple Range Test for FRAPTxg**

NOTE: This test controls the Type I comparisonwise error rate. not the experimentwise error

rate.

Alpha 0.05

Error Degrees of Freedom 8

Error Mean Square 0.068242

Number of Means 2 3 4

Critical Range .4919 .5126 .5241

Means with the same letter are not significantly different.

**Duncan Grouping Mean N Whey**

**A 20.6800 3 0**

**B 9.0300 3 5**

**B 8.9467 3 2.5**

**B 8.5367 3 10**

**Duncan's Multiple Range Test for ABTSTxg**

NOTE: This test controls the Type I comparisonwise error rate. not the experimentwise error

rate.

Alpha 0.05

Error Degrees of Freedom 8

Error Mean Square 0.12775

Number of Means 2 3 4

Critical Range .6730 .7013 .7171

Means with the same letter are not significantly different.

**Duncan Grouping Mean N Whey**

**A 17.6733 3 0**

**B 14.8433 3 2.5**

**B 14.6200 3 5**

**C 11.6833 3 10**
