# Supplementary material for: Mixotrophic cultivation of Spirulina platensis in dairy wastewater: Effects on the production of biomass, biochemical composition and antioxidant capacity
Source: PLoS One. 2019 Oct 24;14(10):e0224294. doi: 10.1371/journal.pone.0224294 (PMC6812818; doi:10.1371/journal.pone.0224294)
Supplement: Attachment — Submitted filename: Response to Reviewers.doc [file pone.0224294.s002.doc]

**Mixotrophic cultivation of Spirulina platensis in dairy wastewater: effect on the production of biomass, biochemical composition and antioxidant capacity**

Dear Academic Editor,

Thank you for your consideration of our manuscript entitled " Mixotrophic cultivation of Spirulina platensis in dairy wastewater: effect on the production of biomass, biochemical composition and antioxidant capacity". We are submitting here a revised version of the manuscript and our responses to the comments of the Plos one Editorial Committee:

**Editor's comments:**

1. To enhance the reproducibility of your results, we recommend that if applicable you deposit your laboratory protocols in [protocols.io](http://protocols.io/), where a protocol can be assigned its own identifier (DOI) such that it can be cited independently in the future.

We made the deposit of 3 methods in protocols.io:

1. [**Clarification of cheese whey for microalgae cultivation**](https://www.protocols.io/view/clarification-of-cheese-whey-for-microalgae-cultiv-4t5gwq6)
   [dx.doi.org/10.17504/protocols.io.4t5gwq6](http://dx.doi.org/10.17504/protocols.io.4t5gwq6).
2. [**Mixotrophic batch growth of Spirulina platensis**](https://www.protocols.io/view/mixotrophic-batch-growth-of-spirulina-platensis-4tygwpw)
   [dx.doi.org/10.17504/protocols.io.4tygwpw](http://dx.doi.org/10.17504/protocols.io.4tygwpw).
3. [**Inoculum preparation for Mixotrophic culturing**](https://www.protocols.io/view/inoculum-preparation-for-mixotrophic-culturing-4txgwpn)
   [dx.doi.org/10.17504/protocols.io.4txgwpn](http://dx.doi.org/10.17504/protocols.io.4txgwpn).

2. Journal Requirements:

2.1. Please ensure that your manuscript meets PLOS ONE's style requirements, including those for file naming. The PLOS ONE style templates can be found at <http://www.journals.plos.org/plosone/s/file?id=wjVg/PLOSOne_formatting_sample_main_body.pdf> and <http://www.journals.plos.org/plosone/s/file?id=ba62/PLOSOne_formatting_sample_title_authors_affiliations.pdf>.

We checked our manuscript according to PLOS ONE's style requirements and adapted it to the requirements style when it was necessary

2.2. Thank you for stating the following in the Acknowledgments Section of your manuscript:"The authors gratefully acknowledge the CAPES (Coordenação de Aperfeiçoamento de Pessoal de Nível Superior) for financial support and Laboratório de Nutrição Animal from the Federal University of Rio Grande do Norte (EAJ/UFRN) for chemical analysis and LARBIM (Laboratório de Ambientes Recifais e Biotecnologia com Microalgas) from the
Federal University of Paraíba for biochemical analysis and for the facilities." We note that you have provided funding information that is not currently declared in your Funding Statement. However, funding information should not appear in the Acknowledgments section or other areas of your manuscript. We will only publish funding information present in the Funding Statement section of the online submission form. Please remove any funding-related text from the manuscript and let us know how you would like to update your Funding Statement. Currently, your Funding Statement reads as follows:"The funders had no role in study design, data collection and analysis, decision to publish, or preparation of the manuscript."

The text of Acknowledgements section was muddy. We have rewritten it as follows:

The authors gratefully acknowledge the Laboratório de Nutrição Animal from the Universidade Federal do Rio Grande do Norte for chemical analysis and the Laboratório de Ambientes Recifais e Biotecnologia com Microalgas from the Universidade Federal da Paraíba for biochemical analysis and for the facilities.

2.3. Please amend your list of authors on the manuscript to ensure that each author is linked to an affiliation. Authors’ affiliations should reflect the institution where the work was done (if authors moved subsequently, you can also list the new affiliation stating “current affiliation:” as necessary).

The affiliation of Bruna M. E. Chagas author was muddy. We have rewritten it as follows:

Bruna M. E. Chagas^1¶#a^, ^1^Agricultural School of Jundiaí, Federal University of Rio Grande do Norte, Natal, RN, Brazil.

^#a^Current Address: Infrastructure Superintendence, Federal University of Rio Grande do Norte, Natal, RN, Brazil.

3. Additional Editor Comments:
3.1. The manuscript has no page neither line numbers. Reviewers have difficulties to track and manage the manuscript if page and line numbers do not exist.

We agree with no page neither line numbers in the manuscript may be confusing for the reviewers. We added page and line numbers to the manuscript.

3.2. The text has too many paragraphs, especially in the Introduction section. Can you please compact it? I also noticed excessive number of references.

We agree with the text has too many paragraphs and references in the manuscript. We reduced paragraphs from the Introduction and Results and Discussion. The references were reduced from 75 to 59.

3.3. It is a common practice to use abbreviation of the species genus, such as *S. platensis*, once you already give the full species binomial name in the first mention.

The name *Spirulina platensis* was abbreviated to *S.platensis* in the text body of the manuscript.

3.4. While revising your submission, please upload your figure files to the Preflight Analysis and Conversion Engine (PACE) digital diagnostic tool, <https://pacev2.apexcovantage.com/>. PACE helps ensure that figures meet PLOS requirements. To use PACE, you must first register as a user. Registration is free. Then, login and navigate to the UPLOAD tab, where you will find detailed instructions on how to use the tool. If you encounter any issues or have any questions when using PACE, please email us at [figures@plos.org](mailto:figures@plos.org). Please note that Supporting Information files do not need this step.

We uploaded our figure files to the Preflight Analysis and Conversion Engine (PACE) digital diagnostic tool as suggested.
